# Comparison of Office-Based Physician Participation in Medicaid Managed Care and Health Insurance Exchange Plans in the Same US Geographic Markets

**DOI:** 10.1001/jamanetworkopen.2020.2727

**Published:** 2020-04-13

**Authors:** Jacob Wallace, Anthony Lollo, Chima D. Ndumele

**Affiliations:** 1 Yale School of Public Health, New Haven, Connecticut

## Abstract

**Importance:**

Several recent policy proposals have sought to expand the role of Medicaid in providing health insurance for low-income Americans, but there is little recent information on how physician participation in Medicaid compares with alternative forms of coverage for low-income Americans.

**Objective:**

To compare the number of office-based physicians included in Medicaid managed care and health insurance exchange plans that operate in the same geographic markets.

**Design, Setting, and Participants:**

This cross-sectional study used administrative data from physician network directories and survey data from office-based physicians for Kansas, Nebraska, New York, Tennessee, and Washington. The number of participants totaled 67 057 office-based physicians in the 5 sample states. Data were collected and analyzed from May 2018 to June 2019.

**Exposures:**

Physician participation in a Medicaid managed care or health insurance exchange plan network.

**Main Outcomes and Measures:**

The percentage of office-based physicians in a county who indicated during a phone survey that they participated in Medicaid; the percentage of office-based physicians in a county who participated in each Medicaid managed care and health insurance exchange plan network; and the percentage of office-based physicians in a county who participated in at least 1 Medicaid managed care plan or, separately, at least 1 health insurance exchange plan.

**Results:**

Of the 67 057 office-based physicians in our sample, 49 983 reported in a telephone survey that they accepted Medicaid. This survey-based measure undercounted the percentage of physicians participating in Medicaid by 5.2% (95% CI, 2.3%-8.1%; *P* < .001) relative to a measure based on physician network directories. Medicaid managed care physician networks covered a median (interquartile range) of 63.4% (48.0%-81.3%) of office-based physicians compared with health insurance exchange physician networks, which covered 51.0% (31.0%-70.5%). In adjusted analyses, Medicaid managed care plans covered 6.2% (95% CI, 3.2%-9.3%, *P* < .001) more office-based physicians than health insurance exchange plans operating in the same counties. In the states where the same insurers participated in both markets (New York, Tennessee, Washington), the Medicaid managed care physician networks were 6.5% (95% CI, 3.2%-9.8%, *P* < .001) larger than the health insurance exchange networks offered by the same insurer.

**Conclusions and Relevance:**

In this cross-sectional study of physician network data, Medicaid managed care physician networks included more office-based physicians than the physician networks of health insurance exchange plans operating in the same geographic markets. This suggests that Medicaid remains a viable option for expanding coverage in the United States.

## Key Points

**Question:**

How does the percentage of office-based physicians who participate in Medicaid compare with participation in health insurance exchange plans?

**Findings:**

In this cross-sectional study of 67 057 office-based physicians in 5 states, Medicaid managed care plans included more physicians than health insurance exchange plans in the same geographic markets.

**Meaning:**

These findings indicate that physicians are likelier to participate in Medicaid physician networks than previously believed, with important implications for the ongoing debate about the role of Medicaid in expanding health insurance and reforming the US health care system.

## Teaser

This cross-sectional study examines state and county physician network data sets to compare physician participation in Medicare networks with health insurance exchange networks.

## Introduction

Medicaid is now the largest single insurer in the United States,^1^ providing coverage for approximately 1 in 5 Americans. Policy makers continue to debate the merits of expanding it further, either through expansions or proposals that would allow low-income individuals to buy into Medicaid,^2,3,4^ but these efforts have been stymied by widely cited concerns about whether the program offers adequate access to physicians and hospitals.^5,6,7,8^ Historically, Medicaid fee-for-service reimbursement rates have been lower than the rates paid to physicians by Medicare or commercial insurers,^9^ limiting physician participation in the program.^10,11,12,13^ However, recent evidence indicates access may be improving in Medicaid, with recipients reporting similar levels of satisfaction as commercial populations,^14^ suggesting a reexamination of access is warranted.

Some policy observers have linked changes in access in Medicaid to the rapid uptake of managed care across states. Currently, more than 80% of Medicaid enrollees receive care through managed care organizations (MCOs),^15^ private health plans that limit patients to a restricted set of physicians and hospitals. This model, known as Medicaid managed care, represents a departure from Medicaid fee-for-service, where states offer contracts to all physicians willing to accept Medicaid reimbursement rates. As a result, for most Medicaid beneficiaries the number of physicians participating in their health plan is a function of the size of the health plan physician networks (hereafter referred to as *physician networks*) offered by each MCO rather than the percentage of physicians willing to accept fee-for-service Medicaid reimbursement rates.^16^ Given the prevalence of Medicaid managed care, a clearer understanding of how MCO physician networks compare to physician networks offered on the health insurance exchanges (HIX) is warranted. This is especially important as states grapple with whether to expand health insurance coverage to low-income, nonelderly Americans through private or public mechanisms.^17^

In this study, we examined the number of physicians participating in Medicaid in several ways. First, we examined how survey-based measures of physician participation in Medicaid, the traditional approach to measuring physician participation, compare with measures of physician participation based on a novel data set of MCO physician network directories obtained directly from state Medicaid agencies. Second, we used physician network directory data to compare the size of MCO and HIX plan networks. We compared 2 distinct measures of physician network size: (1) what percentage of physicians participate in at least 1 MCO vs at least 1 HIX plan, and (2) the size of MCO and HIX networks at the individual plan level, since these are the networks that ultimately determine the set of physicians that are in-network for patients based on the plans they’ve chosen.

## Methods

### Overview

This study, conducted from May 2018 to June 2019, was approved by the institutional review board at Yale Medical School. The requirement for informed consent was waived because participation involved no more than minimal risk to the study participants. The confidentiality of individual practices has been protected. This study follows the Strengthening the Reporting of Observational Studies in Epidemiology (STROBE) reporting guidelines for cross-sectional studies.

### Data

The primary source of data for this study was the physician network directories for MCOs and HIX plans operating in Kansas, Nebraska, New York, Tennessee, and Washington. We measured physician network size at the county level, where we have data on the set of practicing physicians that participate in each Medicaid and HIX network (eTable 1 in the Supplement).

We obtained Medicaid network data directly from the states that included lists of the physicians under contract within its MCO network. We identified the set of counties MCOs participated in using publicly available documentation from each state. We linked the Medicaid network data with information from Vericred Solutions Inc, provided with support from the Robert Wood Johnson Foundation. The Vericred data contained HIX physician networks as of August 1, 2017, obtained either online or from machine-readable physician directories made available by HIX insurers. The data, which we linked at the National Provider Identifier level to our Medicaid network data, have been used in prior research on physician networks.^18,19,20^ To determine which counties each HIX plan operates in, we used the Health Insurance Exchange Compare data set. We only included network-associated plans that were actively marketed in 2017 on a state or federal marketplace. For HIX insurers that offered multiple plans that shared a network, we included the unique network only once. To construct our final sample we excluded a small number of networks where data quality was a concern. We merged the MCO and HIX physician network directories to health care information services firm SK&A’s Office-Based Physician Database (SK&A), a phone-based survey that identifies whether physicians are in active practice and includes their answer to the question “Do you accept Medicaid (yes or no)?” (eAppendix 1 in the Supplement). We restricted our sample to office-based physicians and removed geriatric specialties, since the Medicaid program generally serves as a primary source of coverage for individuals aged 0 to 64 years.

### Variables

Our primary outcome was an assessment of the size of Medicaid managed care and HIX physician networks. We constructed 3 measures of network size. First, we measured the percentage of office-based physicians in a county who answered “yes” to the question “Do you accept Medicaid?” in the SK&A data. Second, we measured the mean percentage of office-based physicians in a county covered by Medicaid managed care and HIX physician networks in that county. Third, we measured the percentage of office-based physicians who participate in at least 1 Medicaid managed care plan in a county and the percentage of office-based physicians who participate in at least 1 HIX plan in a county.

We constructed county-level covariates from several sources. From the 2010 United States Census, we obtained each county’s nonelderly population and racial composition. County-level poverty rates were obtained from the Area Health Resources File. From the Kaiser Family Foundation, we obtained data on health insurance coverage rates and the percentage of Medicaid recipients in managed care in 2017.

### Statistical Analysis

In unadjusted analyses, we presented our measures of physician network size by state, county geographic designation, and physician specialty. We used multivariable regression to attempt to adjust for county using a dummy variable for each county in ordinary least squares regression models of the following form: Y_pc_ = β_0_ + β_1_Medicaid_pc_ + γ_c_ + ε_pc_, where the subscript *p* denotes a plan and *c* denotes a county. The independent variable *Medicaid_pc_* is an indicator that plan *p* offered in county *c* was a Medicaid (rather than HIX) plan. In our adjusted specification, we include dummy variables for each county, *γ_c_*, which attempt to adjust for unobserved factors at the county level so that our estimates can be thought of as comparing the size of Medicaid and HIX physician networks offered within the same county. Standard errors were clustered at the county level and results are reported with 95% confidence intervals and 2-tailed *P* values. We weighted regressions by the county proportion of a state’s population (eAppendix 2, eFigure 1 in the Supplement). To assess the association between the size of Medicaid and HIX physician networks at the state level, we used the Pearson correlation coefficient (denoted by ρ).

Sensitivity analyses tested the robustness of our results to alterations in the statistical model, including the addition of dummy variables for each insurer so that our estimates can be thought of as comparing the size of Medicaid and HIX physician networks offered by the same insurer within the same county.

### Results

#### Population

Our final sample included 2642 physician network–county pairs from 102 physician networks operated by 33 unique issuers in 370 counties in our 5 sample states (eTable 2 in the Supplement). The distribution of physician specialties was qualitatively similar across sample states (eTable 3 in the Supplement). Our sample states were similar to the national average in their demographic characteristics with the exception that, by design, the percentage of the Medicaid population in managed care (89.98%) was higher than average (Table 1).

**Table 1.** Sample State Characteristics

Characteristic	Sample States, %						Rest of states, total, %^a^	Difference (95% CI)	*P* value
	Kansas	Nebraska	New York	Tennessee	Washington	Total			
No. of counties^a^	105	93	62	95	39	78.80	61.02	17.78 (−9.04 to 45.59)	.19
Demographic characteristics^b^									
In poverty	12.27	11.30	14.78	15.98	11.35	13.14	13.62	−0.49 (−1.75 to 0.78)	.45
Nonwhite	13.32	10.65	29.66	21.72	18.84	18.84	20.63	−1.79 (−6.24 to 2.65)	.43
Geographic designation^b^									
Metropolitan population^c^	67.65	64.96	92.85	77.39	90.12	78.59	75.91	2.68 (−4.30 to 9.67)	.45
Micropolitan population^c^	19.20	17.43	5.13	12.99	7.85	12.52	13.79	−1.27 (−6.22 to 3.67)	.61
Nonmetropolitan population^c^	13.14	17.62	2.03	9.62	2.03	8.89	10.29	−1.41 (−4.52 to 1.70)	.37
Medicaid coverage^a^									
Medicaid	14	13	26	21	21	19.00	19.87	−0.87 (−5.56 to 3.83)	.71
Medicaid in managed care	95.5	99.4	74.0	92.6	88.3	89.98	73.28	16.70 (6.05 to 27.35)	.003
Other health insurance coverage^a^									
Employer coverage	55	56	49	48	52	52.00	49.69	2.31 (−1.01 to 5.64)	.17
Non-group coverage	7	8	6	6	6	6.60	6.36	0.24 (−0.62 to 1.11)	.57
Medicare	14	13	12	15	13	13.40	14.27	−0.87 (−1.98 to 0.24)	.12
Uninsured	9	9	6	9	6	7.80	8.29	−0.49 (−2.13 to 1.16)	.55

^a^ Data available at the state level. The mean for “Sample states” (5 states) and “Rest of states” (45 states), as well as the “Difference” columns, computed without weights.

^b^ Data available at the county level. The weighted mean for “Sample states” and “rest of states,” as well as the “Difference” columns, computed weighting by the county proportion of a state’s population.

^c^ Metropolitan population describes the share of the population living in counties with urbanized areas of 50 000 or more population, micropolitan describes counties with urban clusters of at least 10 000 population but less than 50 000 population, and nonmetropolitan is the share of the population living in counties without a micropolitan or metropolitan area.

#### Measuring Participation in Medicaid Managed Care

**Figure 1.**
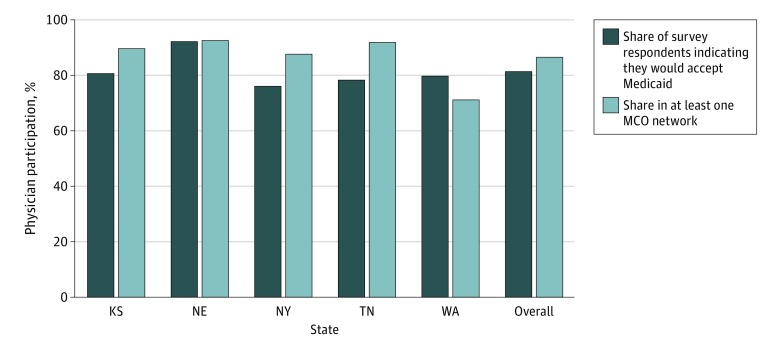
Measuring Physician Participation in Medicaid Using Physician Surveys vs Medicaid Managed Care Network Directories ^a^MCO indicates managed care organization.

Of the 67 057 office-based physicians who met our inclusion criteria, 49 983 reported in a telephone survey that they accepted Medicaid. The percentage of office-based physicians who accepted Medicaid per survey-based reports of participating physicians was 5.2% (95% CI, 2.3%-8.1%; *P* < .001) lower than the percentages that were listed in at least 1 MCO physician network (eTable 4 in the Supplement). This pattern held for Kansas, New York, and Tennessee, but the opposite was true in Washington (Figure 1).

#### Participation in Medicaid Managed Care and the Health Insurance Exchanges

**Figure 2.**
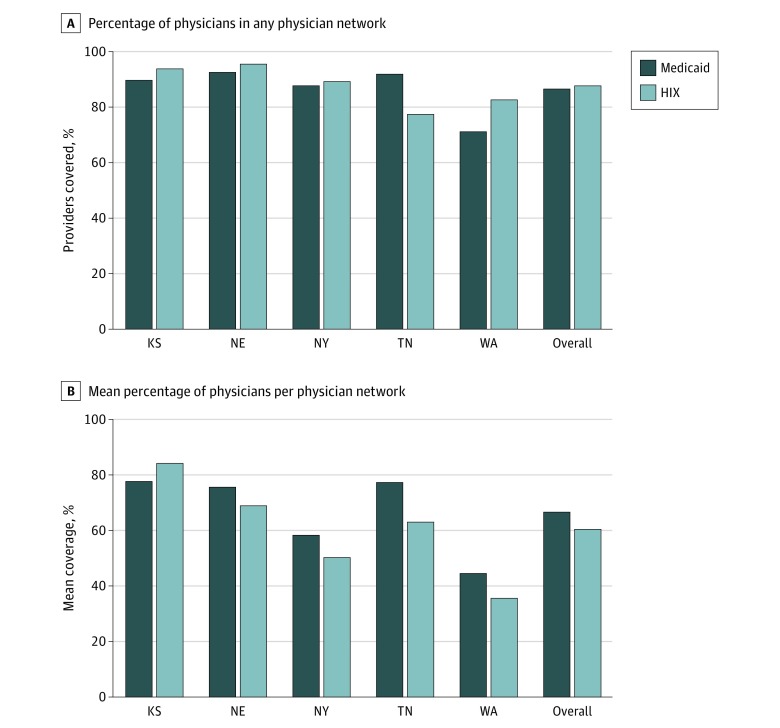
Comparing Medicaid and Health Insurance Exchange Network Size by State ^a^HIX indicates health insurance exchange.

Figure 2 compares the size of Medicaid managed care and HIX physician networks by state. In our study sample, we found that a median (interquartile range [IQR]) of 87.7% (84.7%-93.9%) of office-based physicians participated in at least 1 HIX plan, an estimate that closely mirrors prior work (Table 2).^18^ A similar, but lower, median (IQR) percentage of physicians (86.6% [78.7%-93.1%]) participated in at least 1 MCO physician network. This result masks considerable heterogeneity. In 3 of our 5 states (Nebraska, New York, Washington), a higher percentage of office-based physicians participated in the HIX than Medicaid managed care. We also examined participation in Medicaid managed care and the HIX by physician specialty, based on the difference between participation in at least 1 HIX physician network relative to at least 1 Medicaid physician network. We found a higher percentage of psychiatrists (7.1% [95% CI, 2.7%-11.5%]; *P* = .002) and obstetrician-gynecologists (2.1% [95% CI, 0.1%-4.1%]; *P* = .04) participated in HIX networks relative to Medicaid. These findings were robust to the inclusion of county dummy variables to adjust for potential differences in where MCOs and HIX plans operate.

**Table 2.** Physician Network Size in Medicaid and the Health Insurance Exchanges

State or Characteristic	Mean physicians per network^a^					Physicians in any network^a^				
	Participation in Medicaid, %	Participation in HIX, %	Adjusted difference (95% CI), %^b^	*P* value	No.	Participation in Medicaid, %	Participation in HIX, %	Adjusted difference (95% CI), %^b^	*P* value	No.
Overall	63.4	51.0	6.2 (3.2 to 9.3)	<.001	2462	86.6	87.7	−1.1 (−3.7 to 1.5)	.41	740
States										
Kansas	77.7	84.3	−7.6 (−12.4 to −2.9)	.002	502	89.6	93.8	−4.1 (−8.8 to 0.5)	.08	200
Nebraska	75.6	68.8	3.8 (−5.5 to 13.1)	.40	413	92.6	95.5	−2.9 (−5.0 to −0.8)	.007	152
New York	58.3	50.2	10.7 (7.7 to 13.7)	<.001	816	87.7	89.2	−1.5 (−2.9 to −0.1)	.03	124
Tennessee	77.3	63.1	12.6 (6.2 to 19.0)	<.001	402	91.9	77.4	14.5 (9.6 to 19.5)	<.001	190
Washington	44.4	35.6	6.3 (0.7 to 11.9)	.03	329	71.1	82.6	−11.5 (−13.9 to −9.1)	<.001	74
Urban designation										
Metro	60.5	48.0	7.5 (4.3 to 10.8)	<.001	1077	84.8	86.6	−1.8 (−5.0 to 1.5)	.29	266
Micro	75.1	72.7	−2.8 (−6.7 to 1.2)	.16	487	91.0	90.7	0.3 (−2.1 to 2.7)	.81	146
Nonmetro	82.6	79.1	−1.5 (−5.1 to 2.1)	.40	898	91.6	93.1	2.9 (0.0 to 5.8)	.05	328
Physician specialty										
Primary care	65.1	53.2	5.4 (1.8 to 9.1)	.004	2447	88.6	90.5	−2.0 (−4.0 to 0.1)	.06	734
Cardiology	77.8	59.3	13.6 (6.9 to 20.3)	<.001	1400	95.2	94.5	0.8 (−1.1 to 2.7)	.44	348
Endocrinology	69.9	52.9	13.2 (7.9 to 18.6)	<.001	756	92.6	89.5	3.1 (−0.7 to 7.0)	.11	160
Obstetrician- gynecologist	71.1	56.8	9.6 (3.0 to 16.3)	.005	1375	92.1	94.3	−2.1 (−4.1 to −0.1)	.04	332
Oncology	73.8	56.4	12.4 (6.8 to 18.0)	<.001	1144	94.6	90.7	3.8 (1.2 to 6.5)	.005	274
Psychiatry	46.8	41.4	1.0 (−2.4 to 4.4)	.55	1314	71.6	78.7	−7.1 (−11.5 to −2.7)	.002	300
Surgery	66.7	56.1	6.2 (1.8 to 10.5)	.006	1699	89.0	90.8	−1.8 (−4.0 to 0.6)	.14	442
Other	53.5	40.5	7.8 (4.1 to 11.6)	<.001	1659	79.0	77.3	1.7 (−4.4 to 7.8)	.59	424

Abbreviation: HIX, health insurance exchanges.

^a^ Physicians in any network includes in the numerator all physicians who participate in any of the Medicaid or HIX physician networks operating in a county, with the denominator as all physicians who met our inclusion criteria in that county. It is a county-level measure. The mean percentage of physicians per network includes in the numerator all physicians in a particular Medicaid or HIX physician network operating in a county with the denominator as all physicians who met our inclusion criteria in that county. It is a plan-level weighted average of Medicaid and HIX in each county.

^b^ Adjusted for county dummy variables.

#### Size of Individual Medicaid Managed Care and HIX Physician Networks

In 2017, the mean Medicaid managed care physician network covered 63.4% (95% CI, 48.0%-81.3%; P < .001) of office-based physicians as compared with the average HIX physician network, which covered 51.0% (IQR 31.0%-70.5%) of office-based physicians (Figure 2). On an adjusted basis, Medicaid managed care physician networks covered 6.2% (95% CI, 3.2%-9.3%; *P* < .001) more office-based physicians than HIX physician networks in the same counties (Table 2). When we compared Medicaid managed care and HIX physician networks in metropolitan and nonmetropolitan counties, we found that the difference in size is largest in metropolitan areas, where Medicaid managed care physician networks covered 7.5% (95% CI, 4.3%-10.8%; *P* < .001) more office-based physicians. Medicaid managed care physician networks, on average, include more physicians even when comparing physician networks offered in the same county by the same insurer (eFigure 2 in the Supplement). We tested this formally by estimating a model with county and insurer dummy variables in the 3 states where insurers participate in both markets. Managed care physician networks covered 6.5% (95%, 3.2%-9.8%; *P* < .001) more office-based physicians than HIX physician networks offered by the same insurer (eTable 5 in the Supplement). Insurers participating in both Medicaid and the HIX included more physicians in their physician networks than insurers participating in only 1 of those markets (eFigure 3 in the Supplement).

Similar to prior work,^18,19^ we found large differences across states in the size of the Medicaid and HIX physician networks offered. There was a strong correlation (ρ = 0.92) at the state level between Medicaid managed care and HIX network size. Physician networks in less urban states (Kansas, Nebraska, Washington) covered a greater percentage of physicians. In subanalyses by specialty, Medicaid managed care physician networks included more physicians than HIX physician networks for all but 1 specialty (psychiatry).

## Discussion

This study compared physician participation in Medicaid managed care and the HIX. We found evidence that traditional, survey-based approaches to measuring the number of physicians in Medicaid (ie, tabulating the percentage of physicians that say in telephone surveys that they accept Medicaid) conflict with measures based on physician network directory data. Furthermore, we found that no simple explanation (or adjustment) could reconcile the 2 measures. One possibility is that not all physicians who contract with private, Medicaid managed care plans identify as accepting Medicaid. This finding highlights the importance of incorporating physician network data into measures of physician participation as the percentage of Medicaid recipients in managed care continues to grow.

Using physician network directory data, we examined the relative size of Medicaid managed care and HIX physician networks. We did not find evidence that physicians are more likely to participate in at least 1 MCO than at least 1 HIX plan. On the other hand, the average MCO physician network covers a much larger percentage of office-based physicians than the average HIX physician network. This pattern holds for both primary care and specialty physicians, and is robust to comparisons of the physician networks within insurers that participate in both. Despite being less likely to participate in the average HIX physician network than the average Medicaid physician network, physicians were equally likely to participate in at least 1 HIX physician network as they were to participate in at least 1 Medicaid physician network. This is partially due to HIX plans being more numerous at the county level, as well as MCO physician networks overlapping more across plans than HIX networks (eFigure 4, eTable 6, eAppendix 3 in the Supplement). Given prior evidence that the Medicaid population is served by a concentrated set of physicians,^21^ the differences in overlap across plans between Medicaid and the HIX remain an important area for future work.

One implication of our findings is that only measuring whether physicians participate in at least 1 physician network at the payer level (ie, Medicaid vs HIX) may not capture important differences in physician network size at the plan level (a particular Medicaid vs a particular HIX plan). For example, broader participation at the payer level in the HIX relative to Medicaid may make it likelier that HIX consumers can find plans that include their usual sources of care.^22,23^ However, this relies on consumers navigating a large number of plan choices and complex features, such as physician network size. Prior work suggests consumers will struggle when faced with such choices,^24,25^ although there is evidence consumers take network size into account when selecting plans.^26,27^ Given that consumers value larger physician networks, and may not be fully informed about their future health care needs, it is also important to measure the size of physician networks at the plan level, where network restrictions ultimately bind and limit consumers to a set of contracted physicians.

Our findings also have implications for the regulation of physician networks. For most Medicaid recipients, the number of physicians included in their network is now a function of the size of the physician networks offered by plans participating in Medicaid managed care. Network size is shaped by how states regulate Medicaid managed care, particularly in how they set network adequacy standards—rules for how many and what types of physicians plans must include in their networks. These standards vary widely by state. Theoretical work on health care markets suggests that strict network adequacy standards will limit the flexibility of plans to build high-value networks and negotiate for discounts.^28^ Insufficient network adequacy standards, however, raise the specter of substandard access and prompt concerns that networks may be intentionally designed to avoid the sickest patients.^29,30^ Our findings indicate that Medicaid networks may not be as narrow as once thought.

Our work also contributes to a growing base of evidence that access in Medicaid may be better than previously believed, with important implications for the ongoing debate about how to reform the US health care system. Historical survey data and audits of physicians tended to find reduced access in Medicaid relative to other insurance types.^31,32^ However, recent surveys found that Medicaid recipients report comparable levels of health care satisfaction and, for example, experience similar rates of low-value care to those with other forms of coverage.^14,33,34^ Our findings offer one possible explanation for this—the number of physicians in Medicaid is now largely a function of the size of the physician networks offered by participating plans, networks that appear comparable to, if not broader than, what is being offered on the health insurance exchanges in the states we studied.

This finding has important implications for the ongoing debate about the role of Medicaid in expanding coverage to the remaining uninsured. While roughly a third of states have opted not to expand Medicaid via the Affordable Care Act, Medicaid buy-in and other Medicaid expansion proposals are being debated at the state and federal level.^2,3,4^ Typically, these proposals laud the efficiency of the Medicaid program while raising concerns about whether there is substandard access to physicians and hospitals. Our findings suggest that concerns about limited physician networks in Medicaid may be overstated, and that Medicaid physician networks may include more physicians than plans on the health insurance exchanges.

### Limitations

Our study has several limitations. First, our study is based on a sample of 5 states in 2017, for which Medicaid managed care and HIX physician network information was available. In Table 1 we demonstrate that our sample states are similar to the rest of the nation, although our conclusions may not apply to all states and time periods. Second, we measure the size of MCO and HIX physician networks using data from physician network directories. Previous studies have shown that physician network directories may contain inaccurate information.^35^ This is a particular concern if the physician network data from Medicaid is of a different quality than the physician network data from the HIX. We address this concern by standardizing all physician network data using the National Plan and Provider Enumeration System’s National Provider Identifier (NPPES NPI) Registry and limiting our sample to NPIs that merged with the SK&A Office-Based Physician Database. However, differences in data quality may persist. Third, because we did not have access to administrative claims data, we weight each office-based physician equally. However, prior research suggests that physician characteristics (eg, proximity to patients) will affect their relative importance to Medicaid or HIX enrollees.^36^ Furthermore, measuring physician participation with physician network directories may not account for other factors that impact physician access, including physician capacity, reimbursement rates, or administrative burden.^19,37,38,39^

## Conclusions

In this cross-sectional study of 67 057 office-based physicians operating across 5 states in 2017, we present evidence that traditional, survey-based approaches to measuring physician participation in Medicaid undercount participation relative to measures based on physician network directory data. When participation was measured using physician network data, we found more physicians participating in the average Medicaid plan than the average health insurance exchange plan. Our data suggest that Medicaid physician networks include more physicians than previously believed.

## Notes

Supplement. **eFigure 1.** Histograms by State of the County Proportion of a State’s Population **eFigure 2.** The Mean Percentage of Physicians in Medicaid vs HIX Physician Networks for Insurers Operating in Both the Medicaid and HIX Markets **eFigure 3.** The Mean Percentage of Physicians in Physician Networks for Insurers That Operate in Only One Market (Medicaid or HIX) vs Those That Operate in Both Markets **eFigure 4.** Medicaid and Health Insurance Exchange Physician Network Counts and Overlap by State **eTable 1.** List of Insurers by State **eTable 2.** Physician Exclusions **eTable 3.** Distribution of Physician Specialties by State **eTable 4.** Measuring Physician Participation in Medicaid Using Surveys and Medicaid Managed Care Physician Network Directories **eTable 5.** Mean Percentage of Physicians in Physician Networks in Medicaid Minus HIX With and Without Insurer Dummy Variables **eTable 6.** Number of Physician Networks and Physician Network Overlap **eAppendix 1.** Sample Construction **eAppendix 2.** Regression Specifications **eAppendix 3.** If Medicaid Physician Networks Are Larger on Average Why Do Slightly More Physicians Participate in the Exchanges?
