# Error in Abstract and Results

**DOI:** 10.1001/jamanetworkopen.2020.7601

**Published:** 2020-05-07

 In the Original Investigation titled “Comparison of Office-Based Physician Participation in Medicaid Managed Care and Health Insurance Exchange Plans in the Same US Geographic Markets,”^1^ published April 13, 2020, there was an error in the reporting of statistical measures of physician network coverage that appeared in the Abstract and Results sections. These values should have appeared as means with standard deviations instead of medians with interquartile ranges and means with 95% confidence intervals. This article has been corrected.
