# Supplementary material for: Comparison of Office-Based Physician Participation in Medicaid Managed Care and Health Insurance Exchange Plans in the Same US Geographic Markets
Source: JAMA Netw Open. 2020 Apr 13;3(4):e202727. doi: 10.1001/jamanetworkopen.2020.2727 (PMC7154801; doi:10.1001/jamanetworkopen.2020.2727)
Supplement: Supplement. — eFigure 1. Histograms by State of the County Proportion of a State’s Population eFigure 2. The Mean Percentage of Physicians in Medicaid vs HIX Physician Networks for Insurers Operating in Both the Medicaid and HIX Markets eFigure 3. The Mean Percentage of Physicians in Physician Networks for Insurers That Operate in Only One Market (Medicaid or HIX) vs Those That Operate in Both Markets eFigure 4. Medicaid and Health Insurance Exchange Physician Network Counts and Overlap by State eTable 1. List of Insurers by State eTable 2. Physician Exclusions eTable 3. Distribution of Physician Specialties by State eTable 4. Measuring Physician Participation in Medicaid Using Surveys and Medicaid Managed Care Physician Network Directories eTable 5. Mean Percentage of Physicians in Physician Networks in Medicaid Minus HIX With and Without Insurer Dummy Variables eTable 6. Number of Physician Networks and Physician Network Overlap eAppendix 1. Sample Construction eAppendix 2. Regression Specifications eAppendix 3. If Medicaid Physician Networks Are Larger on Average Why Do Slightly More Physicians Participate in the Exchanges? [file jamanetwopen-3-e202727-s001.pdf]

Supplementary Online Content

Wallace J, Lollo A, Ndumele CD. Comparison of office-based physician participation in Medicaid managed care and health insurance exchange plans in the same US geographic markets. *JAMA Netw Open*. 2020;3(4):e202727. doi:10.1001/jamanetworkopen.2020.2727

eFigure 1. Histograms by State of the County Proportion of a State Populations
eFigure 2. The Mean Percentage of Physicians in Medicaid vs HIX Physician Networks for Insurers Operating in Both the Medicaid and HIX Markets
eFigure 3. The Mean Percentage of Physicians in Physician Networks for Insurers That Operate in Only One Market (Medicaid or HIX) vs Those that Operate in Both Markets
eFigure 4. Medicaid and Health Insurance Exchange Physician Network Counts and Overlap by State
eTable 1. List of Insurers by State
eTable 2. Physician Exclusions
eTable 3. Distribution of Physician Specialties by State
eTable 4. Measuring Physician Participation in Medicaid Using Surveys and Medicaid Managed Care Physician Network Directories
eTable 5. Mean Percentage of Physicians in Physician Networks in Medicaid Minus HIX With and Without Insurer Dummy Variables
eTable 6. Number of Physician Networks and Physician Network Overlap
eAppendix 1. Sample Construction
eAppendix 2. Regression Specifications
eAppendix 3. If Medicaid Physician Networks Are Larger on Average Why Do Slightly More Physicians Participate in the Exchanges?

This supplementary material has been provided by the authors to give readers additional information about their work.

© 2020 Wallace J et al. *JAMA Network Open.*

eFigure 1. Histograms by state of the county proportion of a state populations

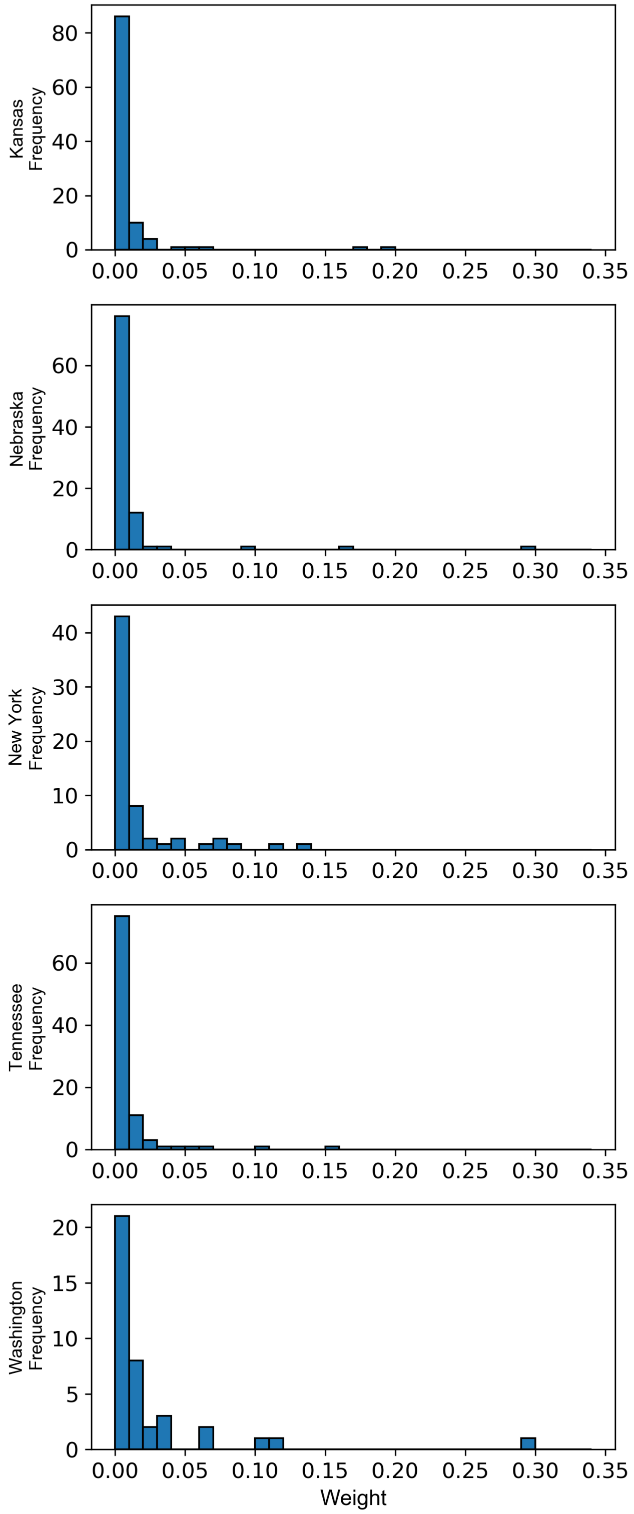

© 2020 Wallace J et al. *JAMA Network Open.*

eFigure 2. The mean percentage of physicians in Medicaid vs HIX physician networks for insurers operating in both the Medicaid and HIX markets

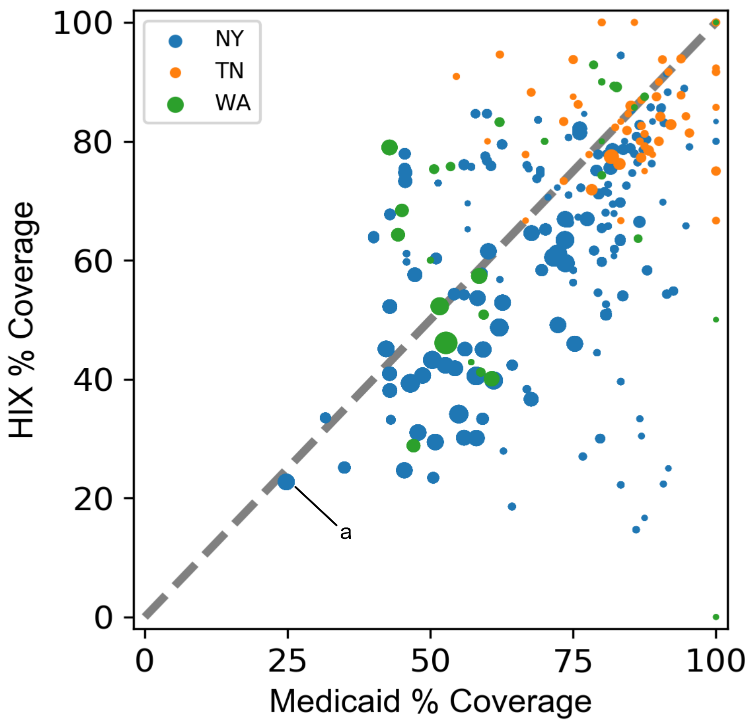

The forty-five degree line is indicated by the thick, dashed gray line.

[a] This point in the figure refers to a county in New York. The x-axis value is the mean percentage of physicians in the Medicaid physician networks of insurers that participate in both markets in that county. The y-axis value is the mean percentage of physicians in HIX physician networks of insurers that participate in both markets in that county.

© 2020 Wallace J et al. *JAMA Network Open.*

eFigure 3. The mean percentage of physicians in physician networks for insurers that operate in only one market (Medicaid or HIX) vs those that operate in both markets

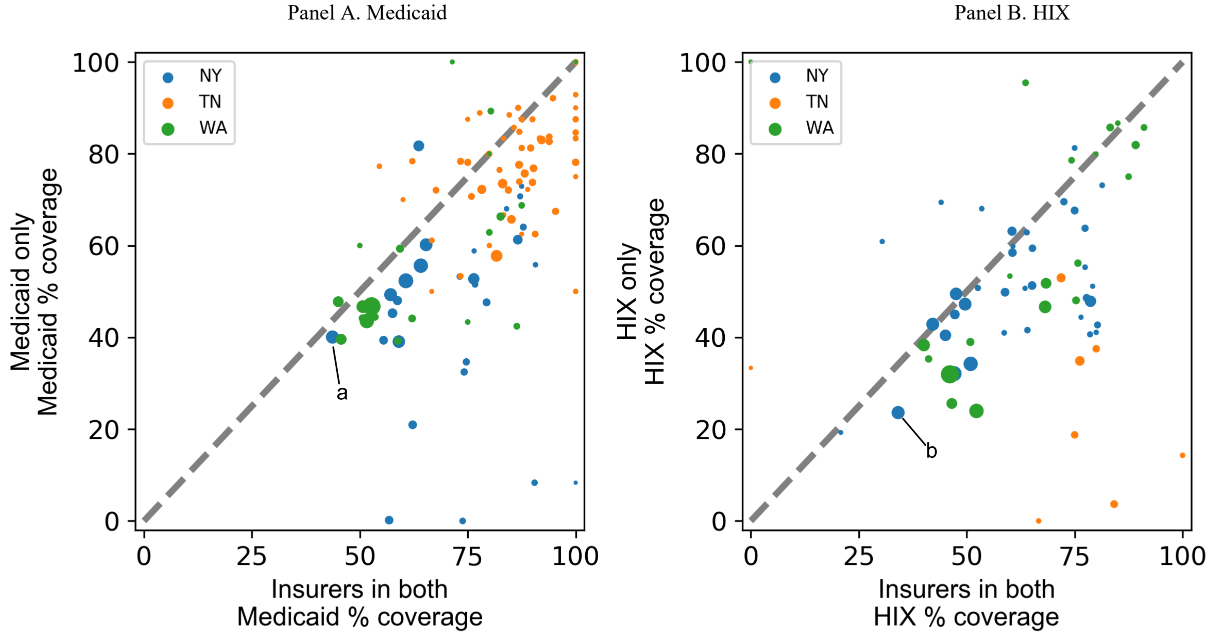

The forty-five degree line is indicated by the thick, dashed gray line.

[a] This point in the figure refers to a county in New York. The x-axis value is the mean percentage of physicians in the Medicaid physician networks of insurers that participate in both markets in that county. The y-axis value is the mean percentage of physicians in Medicaid physician networks of insurers that participate in only Medicaid.

[b] This point in the figure refers to a county in New York. The x-axis value is the mean percentage of physicians in the HIS physician networks of insurers that participate in both markets in that county. The y-axis value is the mean percentage of physicians in HIX physician networks of insurers that participate in only HIX.

© 2020 Wallace J et al. *JAMA Network Open.*

eFigure 4. Medicaid and Health Insurance Exchange physician network counts and overlap by state

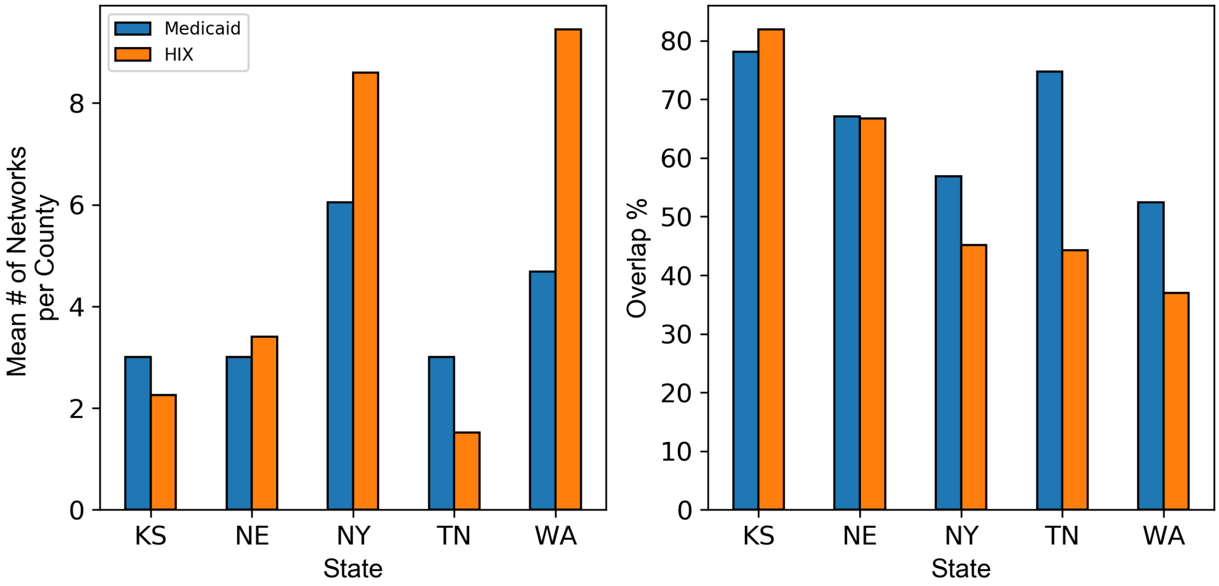

© 2020 Wallace J et al. *JAMA Network Open.*

eTable 1. List of insurers by state

State	Market	Insurer
Kansas	Individual	Medica
	Individual	BlueCross BlueShield of Kansas
	Individual	BlueCross BlueShield of Kansas City
	Medicaid	Amerigroup
	Medicaid	Centene
	Medicaid	UnitedHealthcare
Nebraska	Individual	Medica
	Individual	Aetna
	Medicaid	Centene
	Medicaid	WellCare
	Medicaid	UnitedHealthcare
New York	Individual	MVP Health Plans
	Individual	CDPHP
	Individual	BlueShield of Northeastern New York
	Individual	Empire BlueCross BlueShield
	Individual	EmblemHealth
	Individual	Fidelis Care
	Individual	Independent Health
	Individual	BlueShield of Western New York
	Individual	CareConnect Insurance
	Individual	HealthFirst
	Individual	MetroPlus Health Plan
	Individual	Affinity Health Plan
	Individual	UnitedHealthcare
	Individual	Oscar
	Individual	Excellus
	Individual	Crystal Run
	Medicaid	Fidelis Care
	Medicaid	UnitedHealthcare
	Medicaid	WellCare
	Medicaid	CDPHP
	Medicaid	MVP Health Plans
	Medicaid	YourCare
	Medicaid	HealthNow
	Medicaid	HealthFirst

© 2020 Wallace J et al. *JAMA Network Open.*

State (continued)	Market (continued)	Insurer (continued)
New York	Medicaid	EmblemHealth
	Medicaid	Amerigroup
	Medicaid	Affinity Health Plan
	Medicaid	MetroPlus Health Plan
	Medicaid	Excellus
	Medicaid	Molina
	Medicaid	Independent Health
	Medicaid	Crystal Run
Tennessee	Individual	Humana
	Individual	BlueCross BlueShield of Tennessee
	Individual	Cigna
	Medicaid	Amerigroup
	Medicaid	UnitedHealthcare
	Medicaid	BlueCross BlueShield of Tennessee
Washington	Individual	Community Health Plan of Washington
	Individual	Premera Blue Cross
	Individual	Molina
	Individual	LifeWise Health Plan
	Individual	Ambetter
	Individual	Kaiser Permanente
	Individual	BridgeSpan Health Company
	Individual	Regence BlueCross BlueShield
	Medicaid	Community Health Plan of Washington
	Medicaid	Molina
	Medicaid	Centene
	Medicaid	UnitedHealthcare
	Medicaid	Amerigroup

© 2020 Wallace J et al. *JAMA Network Open.*

eTable 2. Physician exclusions

	Medicaid	HIX	Medicaid %	HIX %
**Raw Data**	256,687	1,026,723	100.00	100.00
**Observations Dropped**				
1. SK&A Office-Based Physician Database merge	193,842	959,623	75.52	93.46
2. Remove physicians with missing primary taxonomy code	507	576	0.20	0.06
3. Remove non-MDDO or geriatric specialties	5,588	6,659	2.18	0.65
4. Remove entities	0	0	0	0
5. Remove physicians without a county mapping	0	0	0	0
6. Remove practice location outside of network	408	2,530	0.16	0.25
**Physicians Remaining**	56,342	57,335	21.95	5.58

© 2020 Wallace J et al. *JAMA Network Open.*

eTable 3. Distribution of physician specialties by state

	Percentage of the total office-based physicians in each state in each specialty (%)				
	**KS**	**NE**	**NY**	**TN**	**WA**
Cardiology	5	4.6	5.3	5.1	3.5
Endocrinology	1.1	0.9	1.7	1.3	1.2
OB	5.2	5.7	5.6	5.5	4.8
Oncology	2.9	3.2	3.4	3.4	3.5
Other	24.9	26.3	27.2	26.5	28.3
PC	45.7	43.7	41.1	42.6	44.8
Psychiatry	3.8	3.1	5.8	3.2	3.1
Surgery	11.3	12.5	9.8	12.4	10.7

© 2020 Wallace J et al. *JAMA Network Open.*

eTable 4. Measuring physician participation in Medicaid using surveys and Medicaid managed care physician network directories

	Percentage answering "yes" to accepting Medicaid (1)	Percentage in any MMC network (2)	Mean Percentage per MMC network (3)	Adjusted Difference (2)-(1) (SD)	*P* Value (2)-(1)	Adjusted Difference (3)-(1) (SD)	*P* Value (3)-(1)
**Overall**	81.4	86.6	63.4	5.2 (1.4)	*P*<.001	-15.0 (1.8)	*P*<.001
**States**							
Kansas	80.6	89.6	77.7	9.1 (2.3)	*P*<.001	-2.9 (1.3)	.02
Nebraska	92.2	92.6	75.6	0.4 (1.9)	.85	-16.6 (1.4)	*P*<.001
New York	76.1	87.7	58.3	11.6 (2.1)	*P*<.001	-16.2 (2.1)	*P*<.001
Tennessee	78.3	91.9	77.3	13.6 (1.4)	*P*<.001	-1.0 (1.1)	.37
Washington	79.8	71.1	44.4	-8.6 (2.8)	.004	-35.2 (2.1)	*P*<.001

© 2020 Wallace J et al. *JAMA Network Open.*

eTable 5. Mean percentage of physicians in physician networks in Medicaid minus HIX with and without insurer dummy variables

	Main specification (%)		Specification with insurer dummy variables (%)		N
	Difference (SD)	*P* Value	Difference (SD)	*P* Value	
**Overall (NY, TN, WA)**	9.2 (1.2)	*P*<.001	6.5 (1.7)	*P*<.001	1547
**States**					
New York	10.7 (1.5)	*P*<.001	10.4 (1.1)	*P*<.001	816
Tennessee	12.6 (3.2)	*P*<.001	0.8 (1.3)	.53	402
Washington	6.3 (2.8)	.03	-6.0 (4.4)	.18	329
**Geographic designation**					
Metro	9.6 (1.3)	*P*<.001	6.9 (1.8)	*P*<.001	897
Micro	2.0 (3.1)	.52	3.0 (2.9)	.31	339
Non-metro	5.7 (3.2)	.08	2.7 (2.4)	.28	311
**Physician Specialty**					
Primary Care	8.6 (1.6)	*P*<.001	6.6 (2.2)	.003	1542
Cardiology	19.2 (3.3)	*P*<.001	9.0 (1.3)	*P*<.001	1174
Endocrinology	15.5 (2.7)	*P*<.001	8.4 (2.3)	*P*<.001	691
OB/Gyn	14.7 (3.3)	*P*<.001	8.8 (1.9)	*P*<.001	1146
Oncology	16.7 (2.8)	*P*<.001	8.2 (2.0)	*P*<.001	938
Psychiatry	3.2 (1.6)	.05	9.5 (1.6)	*P*<.001	1141
Surgery	11.5 (1.8)	*P*<.001	5.7 (1.9)	.004	1289
Other	7.1 (1.8)	*P*<.001	5.7 (1.8)	.002	1299

© 2020 Wallace J et al. *JAMA Network Open.*

eTable 6. Number of physician networks and physician network overlap

	Average # of networks in a county					Average network overlap in a county (Jaccard similarity)				
	**Medicaid**	**HIX**	**Adjusted Difference (SD)**	***P* Value**	**N**	**Medicaid**	**HIX**	**Adjusted Difference (SD)**	***P* Value**	**N**
**Overall**	3.9	5.0	-1.10 (0.55)	.05	788	65.9	56.3	8.3 (2.2)	*P*<.001	570
**States**										
Kansas	3.0	2.3	0.75 (0.16)	*P*<.001	210	78.2	81.9	-3.8 (1.5)	.02	200
Nebraska	3.0	3.4	-0.40 (0.55)	.47	186	67.1	68.0	-0.8 (5.3)	.88	140
New York	6.0	8.6	-2.54 (0.75)	.001	124	56.9	45.2	11.7 (3.1)	*P*<.001	118
Tennessee	3.0	1.5	1.48 (0.11)	*P*<.001	190	74.8	44.3	28.5 (6.4)	*P*<.001	44
Washington	4.7	9.4	-4.76 (1.43)	.001	78	52.4	37.0	15.4 (4.3)	.001	68

The measure of network overlap is the Jaccard similarity, which is the percentage of physicians participating in any pair of networks divided by the number of unique physicians participating in either network.

© 2020 Wallace J et al. *JAMA Network Open.*

eAppendix 1. Sample construction

To construct our final sample we started with 104 networks, 30 MMC and 74 HIX, and removed two HIX networks in New York where we had concerns about quality of the network data. In our study sample, 24 non-metro counties (2 in Washington, 5 in Kansas and 17 in Nebraska) had zero office-based physicians whose primary address was located in that county. Any network-county pairs with these counties were excluded from the analyses.

To obtain a standard set of physicians we merged the MCO and HIX physician network directories to the SK&A Office-Based Physician Database. The database contained 76,311 NPIs of active office-based physicians in 2017 with their primary address and the answer to the question "Do you accept Medicaid (yes or no)?" The NPIs that did not merge to the database were removed. A physician's primary taxonomy was determined from the NPPES NPI Registry. Of the physicians that merge to the SK&A database we exclude 0.8% (507/62,845) of Medicaid physicians and 0.9% (576/67100) of HIX physicians who are missing a primary taxonomy code. We kept physicians with a primary taxonomy group of Allopathic & Osteopathic Physicians and excluded physicians who primarily specialize in Geriatric Medicine or Geriatric Psychiatry since they primarily serve the elderly. We make this restriction since the Medicaid program generally serves as a primary source of coverage for the non-elderly, ages 0-64. We assigned a single county to each physician based on their primary address. For ZIP codes that mapped to multiple counties we assigned the physician to the county containing the plurality of addresses for that ZIP code. For each ZIP code, we identified the county containing the plurality of its addresses by using the Housing and Urban Development zip-to-county crosswalk for 2017. We excluded physicians from directories whose primary address was located in a county that was not served by that network (**eTable 2**).[20]

© 2020 Wallace J et al. *JAMA Network Open.*

eAppendix 2. Regression specifications

The analyses presented in Exhibit 3 for the "mean percentage of physicians per network" are based on specifications of the form:

$$Y_{pc} = \alpha + \gamma_c County_c + \beta Medicaid_{pc} + \epsilon_{pc}$$

where p indexes plans, c indexes counties, Y_{pc} is the percentage of office-based physicians in a county that are covered by plan p in county c, $\gamma_c County_c$ is a full set of county fixed effects, and $\beta Medicaid_{pc}$ is an indicator equal to one if a plan is offered in Medicaid managed care and zero if the plan is offered on the HIX, and ϵ_{pc} is a noise term. Each observation is at the plan-county level.

The results for the "percentage of physicians in any network" are based on specifications of the form:

$$Y_{mc} = \alpha + \gamma_c County_c + \beta Medicaid_{mc} + \epsilon_{mc}$$

where m indexes markets, c indexes counties, Y_{mc} is the percentage of office-based physicians in a county that participate in any of the plans in a particular market (i.e. Medicaid managed care and the HIX), $\gamma_c County_c$ is a full set of county fixed effects, and $\beta Medicaid_{mc}$ is an indicator equal to one if the market is Medicaid managed care and zero if the market is the HIX, and ϵ_{mc} is a noise term. Each observation is at the market-county level.

The results presented in **eTable 4** that included insurer dummy variables are based on regressions of the form:

$$Y_{ipc} = \alpha + \gamma_c County_c + \delta_i Issuer_i + \beta Medicaid_{pc} + \epsilon_{ipc}$$

where p indexes plans, c indexes counties, Y_{ipc} is the percentage of office-based physicians in a county that are covered by plan p in county c, $\gamma_c County_c$ is a full set of county fixed effects, $\delta_i Issuer_i$ is a full set of issuer fixed effects, and $\beta Medicaid_{pc}$ is an indicator equal to one if a plan is offered in Medicaid managed care, and ϵ_{pc} is a noise term. Each observation is at the plan-county level.

eAppendix 3. If Medicaid physician networks are larger on average why do slightly more physicians participate in the Exchanges?

We document the number of unique Medicaid managed care and HIX physician networks in each county. For Medicaid managed care, we used publicly-available documentation from each state to identify the number of plans participating in each county. For the HIX, physician networks are listed by rating areas, which were mapped to counties using the HIX crosswalk. However, plans may not be offered throughout an entire rating area. We used the HIX Issuer County Report to include only the physician network-county pairs where the issuer had at least one on-market plan served by that physician network in that county.

We define the overlap between two physician networks at the county-level as the number of physicians practicing in that county that participate in both physician networks divided by the number of unique physicians participating in either physician network, (i.e. the Jaccard similarity). To obtain a measurement at the county-level by payer type, we take the average of all pairwise combinations of networks of the same payer type within the same county.

We explore two interacting hypotheses for why at the plan level Medicaid managed care physician networks cover a higher percentage of office-based physicians than HIX plans but at the county level, fewer physicians participate in Medicaid managed care than in the HIX. First, we document the fact that there tend to be more HIX plans per county operating in our sample states (**eFigure 3**). Pooling data from our sample states, we find that there are 1.10 (0.01 - 2.18) more Exchange plans operating per county in adjusted analyses (**eTable 5**). Hence, even if at the plan level Medicaid managed care physician networks tend to be broader, at the county level more physicians may participate in the HIX simply by virtue of there being more plans. However, this relationship is complicated by the degree of overlap within the Medicaid managed care and HIX markets. If different HIX plans tend to cover the same set of physicians then

© 2020 Wallace J et al. *JAMA Network Open.*

adding additional HIX plans to a county would not increase the percentage of physicians that participate in the HIX at the county level. In our sample, we tend to find the opposite, that there is more overlap across the physician networks within Medicaid managed care than there is across the networks within the HIX (**eFigure 3**). In adjusted analyses across our five states, we find that the overlap within Medicaid physician networks is 8.3 % points (4.0 - 12.6) higher than the overlap within HIX networks (**eTable 5**).

© 2020 Wallace J et al. *JAMA Network Open.*
